# Appraising the causal relationship between thyroid function and rheumatoid arthritis: a two-sample bidirectional Mendelian randomization study

**DOI:** 10.3389/fimmu.2023.1238757

**Published:** 2023-11-28

**Authors:** Peng Gu, Bin Pu, YangCheng Ma, Dan Yue, Qiao Xin, HaiShan Li, Teng Liu, XiaoHui Zheng, ChongZhi Ouyang

**Affiliations:** ^1^ The First Affiliated Hospital, Guangzhou University of Chinese Medicine, Guangzhou, China; ^2^ First School of Clinical Medicine, Guangzhou University of Chinese Medicine, Guangzhou, Guangdong, China; ^3^ College of Integrated Chinese and Western Medicine, Southwest Medical University, Luzhou, Sichuan, China; ^4^ Graduated School, Jiangxi University of Chinese Medicine, Nanchang, Jiangxi, China

**Keywords:** Mendelian randomization, rheumatoid arthritis, hyperthyroidism, hypothyroidism, free thyroxine, thyroid-stimulating hormone

## Abstract

**Background:**

Hypothyroidism and hyperthyroidism are observationally associated with rheumatoid arthritis (RA), but causality is unclear. To evaluate the causal relationship between thyroid function and RA, we conducted a two-Sample bidirectional Mendelian Randomization (MR) study.

**Methods:**

Single nucleotide polymorphisms associated with six phenotypes were selected from the FinnGen biobank database, The ThyroidOmics Consortium database, and the IEU Open GWAS database. For the forward MR analysis, we selected hypothyroidism (N=213,390), Graves’ disease (GD) (N=199,034), other types of hyperthyroidism (N=190,799), free thyroxine (FT4, N=49,269), and thyroid-stimulating hormone (TSH, N=54,288) as the five related thyroid function phenotypes for exposure, with RA (N=58,284) as the outcome. Reverse MR analysis selected RA as the exposure and five phenotypes of thyroid function as the outcome. The Inverse variance weighting (IVW) method was used as the primary analysis method, supplemented by weighted median (WM) and MR-Egger methods. Cochran’s Q test, MR-PRESSO, MR-Egger regression methods, and leave-one-out analysis were employed to assess sensitivity and pleiotropy.

**Results:**

Forward MR evidence indicates that genetic susceptibility to hypothyroidism is associated with an increased risk of RA (OR_Ivw_=1.758, P=7.61×10^-5^). Reverse MR evidence suggests that genetic susceptibility to RA is associated with an increased risk of hypothyroidism (OR_Ivw_=1.274, P=3.88×10^-20^), GD (OR_Ivw_=1.269, P=8.15×10^-05^), and other types of hyperthyroidism (OR_Ivw_=1.141, P=1.80×10^-03^). There is no evidence to support a forward or reverse causal relationship between genetic susceptibility to RA and FT4, TSH.

**Conclusion:**

Our results provide genetic evidence supporting bidirectional causal relationships between thyroid function and RA. These findings inform preventive strategies and interventions targeting RA and thyroid dysfunction.

## Introduction

Rheumatoid arthritis (RA) is a common autoimmune disease that can occur at any age and is more prevalent in females. The global prevalence of RA is approximately 0.5-1% (1, 2). RA is characterized by persistent symmetric polyarticular synovial tissue inflammation, which can destroy articular cartilage and juxtaarticular bone (1, 3), resulting in joint damage, deformity, and physical disability. Many RA patients may also experience extra-articular RA or complications involving the cardiovascular, gastrointestinal, cutaneous, pulmonary, ocular, renal, skeletal, and thyroid systems (4, 5). The pathogenesis of RA is complex and involves the interplay of multiple factors, including abnormal activation of the immune system and sustained inflammatory response with immune cells infiltrating the synovial tissue of the joints (6). Several thyroid diseases, mainly Hashimoto’s thyroiditis (HT) and Graves’ disease (GD), are also mediated by autoimmune mechanisms.

Thyroid dysfunction encompasses two major forms, hyperthyroidism and hypothyroidism, including their overt and subclinical stages. Common causes of thyroid dysfunction include thyroid surgery, radiotherapy, certain medications, iodine deficiency, and various autoimmune diseases. Numerous studies have explored the association between thyroid dysfunction and rheumatologic autoimmune diseases, such as systemic lupus erythematosus (7), Sjögren’s syndrome (8), and RA (9, 10). Among them, we are particularly interested in investigating the relationship between thyroid dysfunction and RA. However, it remains unclear whether there is a causal relationship between thyroid dysfunction and RA, as previous observational studies may be subject to residual confounding, selection bias, and reverse causality. Additionally, conducting randomized controlled trials (RCTs) on thyroid diseases poses challenges regarding sample size and long-term follow-up.

In recent years, Mendelian randomization (MR) has emerged as a valuable method for inferring potential causal relationships between exposures and outcomes, utilizing genetic variants as instrumental variables (IVs). MR studies capitalize on the random allocation of genetic variation, the lack of mutual influence between different traits, and the stability of allele frequencies in the face of disease, thereby mitigating limitations associated with observational studies and randomized controlled trials (11–14).

In this study, we employed publicly available genome-wide association study (GWAS) data and conducted a two-sample bidirectional MR analysis to explore potential causal relationships between hypothyroidism, GD, other types of hyperthyroidism, thyroid-stimulating hormone (TSH), free thyroxine (FT4), and rheumatoid arthritis (RA). Additionally, we aimed to elucidate their interactions and provide novel insights into underlying mechanisms.

## Methods

### Study design and data sources

**Figure 1:**
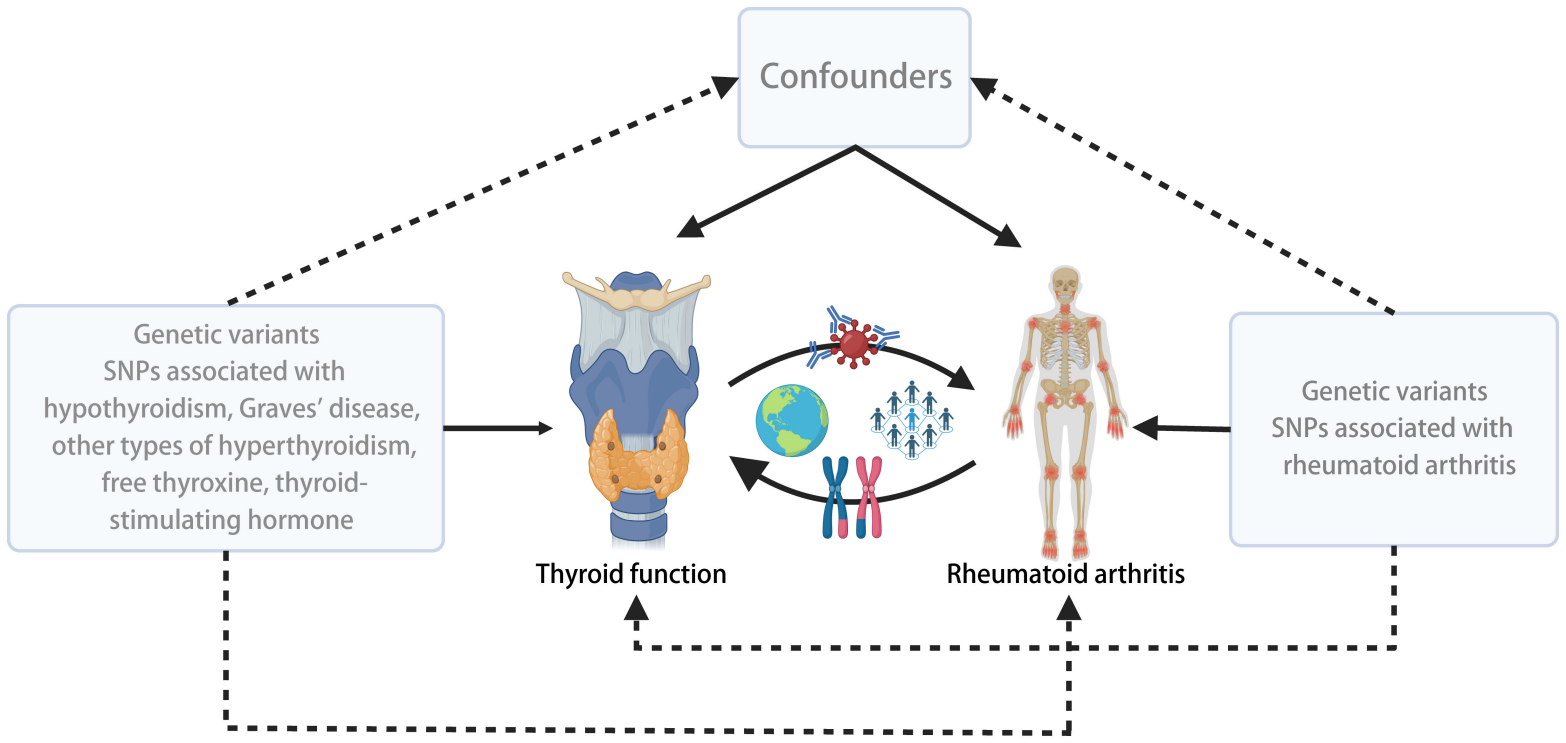
Overall design and flow chart of the present study.

We conducted a two-sample bidirectional MR study using summary data from different GWAS for thyroid function and RA. To minimize potential confounding bias due to population stratification, we restricted our study population to individuals of European ancestry. The flowchart outlining the design of the bidirectional MR study is depicted in **Figure 1**.

Genetic association data related to RA were obtained from the IEU Open GWAS database (https://gwas.mrcieu.ac.uk/), with a sample size of 58,284, including 14,361 RA cases and 43,923 control samples (15). GWAS data for hypothyroidism, GD, and other types of hyperthyroidism were sourced from the FinnGen biobank database (https://www.finngen.fi/en/node/17), comprising 26,036, 2,350, and 3,115 cases, respectively, and 187,684 control samples (16). Summary-level GWAS data for FT4 and TSH were obtained from the ThyroidOmics Consortium database (www.thyroidomics.com), with sample sizes of 54,288 and 49,269, respectively (17). Detailed information regarding the phenotypes is provided in **Supplementary Table 1**.

The datasets used in our study are publicly available, and each GWAS study included has obtained ethical approval from their respective institutional review boards. Our study adheres to the Strengthening the Reporting of Observational Studies in Epidemiology (STROBE) guideline (18).

### IV selection criteria

If the following conditions are met, MR analysis can produce an unbiased estimate of the bidirectional causal relationship between Thyroid function and RA: (1) Genetic IVs are only related to the exposure; (2) IVs are independent of related confounding factors; (3) IVs only affects the outcome. To construct the genetic IVs, we identified single nucleotide polymorphisms (SNPs) that were significantly associated with the exposure based on stringent criteria (P < 5×10^-8^) and independence (r^2^ < 0.001, kb = 10000).

We chose not to use the SNP proxy and set the minimum allele frequency (MAF)to 0.001. In addition, we coordinated the effect alleles between the exposure and outcome data sets, excluding all SNPs with palindromes. To assess the strength of the IVs, we employed the F statistical value, calculated as F = (N-2) * R^2/(1-R^2), where R^2 represents the variance of exposure explained by the genetic instrumental (determined by the effect allele frequency (EAF) and the genetic effect of exposure), and N denotes the sample size (19). The F value greater than 10 indicated a lower risk of weak IV bias (20).

### Statistical analysis

Firstly, we employed three methods, Inverse Variance Weighting (IVW), Weighted Median (WM), and MR Egger, to estimate the causal effects. IVW was the primary analysis method, yielding the highest statistical power when all IVs were valid instruments (21). Subsequently, we employed Cochran’s Q test to assess heterogeneity among the SNPs in the IVW estimation (22). If the heterogeneity test yielded a P-value < 0.05, we utilized the MR-PRESSO method to identify and remove IVs with heterogeneity from the analysis. We screened and excluded SNPs associated with confounding factors using the PhenoScanner database (http://www.phenoscanner.medschl.cam.ac.uk/) (23, 24). The potential confounding factors include smoking, alcohol consumption, body mass index, and educational level. The remaining IVs were then subjected to the IVW method to obtain the final effect estimates. We assessed the presence of horizontal pleiotropy using the MR-Egger intercept test, where an intercept value close to 0 and a P-value > 0.05 suggest no horizontal pleiotropy (25). If heterogeneity persisted, we reported the results from the IVW random-effects model as the primary effect estimate; otherwise, we used the fixed-effects model (26). The criteria for establishing a causal relationship included significant results in the IVW analysis, and the results of WM and MR-Egger analysis are in the same direction as those of the IVW analysis (27–29). Additionally, we conducted the leave-one-out analysis to examine the influence of individual SNPs on the overall causal effect (30). Funnel plots were used to assess the symmetry of selected SNPs, forest plots were employed to evaluate the reliability and heterogeneity of incidental estimates, and scatter plots were used to visualize the effect relationship between exposure and outcome.

In the reverse analysis, we applied the same methods as described above, utilizing an SNP set related to RA to examine the causal effects between RA and Thyroid function, including Hypothyroidism, GD, other types of Hyperthyroidism, FT4, and TSH (**Figure 1**).

The statistical analyses were performed using the R packages “TwoSample MR” and “MR-PRESSO” with R software version 4.3.0.

## Results

### IV selection

In the forward analysis, we obtained 52, 12, 11, 45, and 24 IVs independent of linkage disequilibrium (LD) from hypothyroidism, GD, other types of hyperthyroidism, FT4, and TSH, respectively. In the reverse analysis, 86 SNPs related to the RA were selected as IVs. The F statistical value of each selected IV is greater than 10, indicating that there is unlikely to be a weak IV bias. The information of SNPs on exposure is listed in **Supplementary Tables 2**–**7**.

### Influence of genetically predicted thyroid function on RA

**Figure 2:**
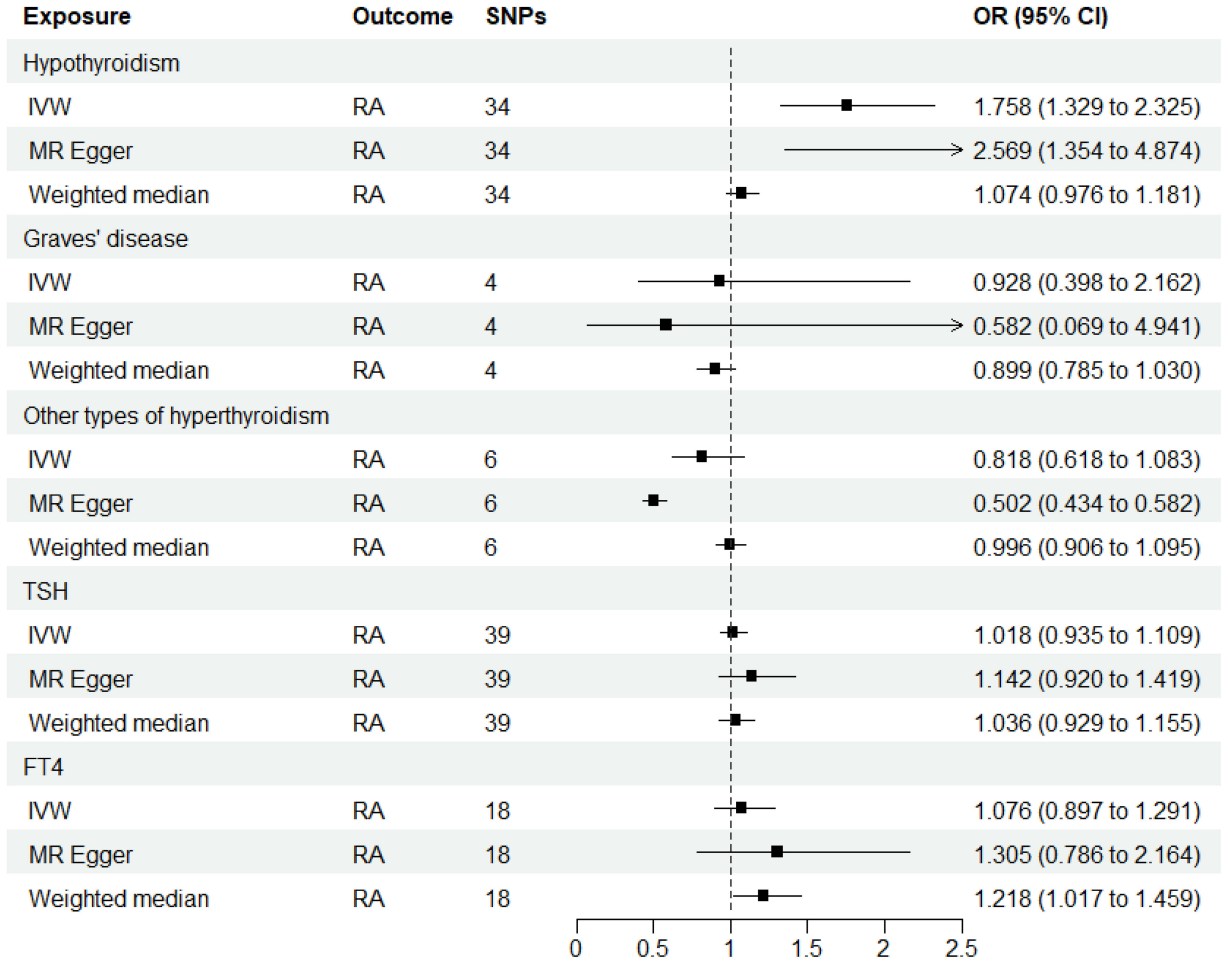
MR estimates for the causal effect of thyroid function on RA. IVW, inverse variance weighted; SNP, single-nucleotide polymorphism; OR, odds ratio; CI, confidence interval; RA, rheumatoid arthritis; TSH, thyroid-stimulating hormone; FT4, free thyroxine.

**Figure 3:**
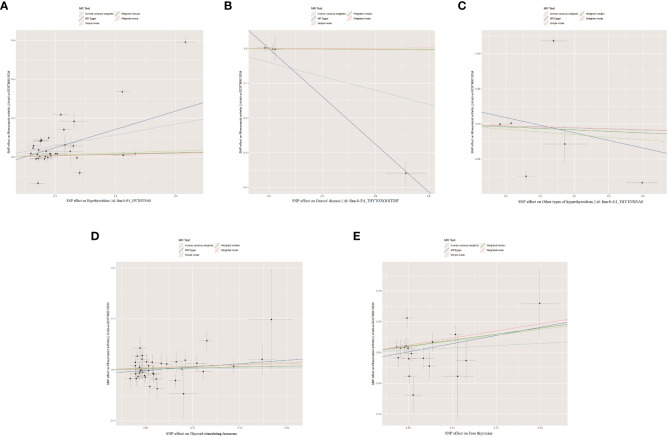
The scatter plots for the causal effect of forward MR analysis. **(A)** Hypothyroidism on rheumatoid arthritis. **(B)** Graves' disease on rheumatoid arthritis. **(C)** Other types of hyperthyroidism on rheumatoid arthritis. **(D)** Thyroid-stimulating hormone on rheumatoid arthritis. **(E)** Free thyroxine on rheumatoid arthritis.

As shown in **Supplementary Tables 2**–**7**, after excluding 4, 1, 2, 4, and 6 palindromic SNPs and 15, 8, 3, 2, and 0 heterozygous SNPs (which may overlap) for hypothyroidism, GD, other types of hyperthyroidism, TSH, and FT4, respectively, we obtained a final set of 34, 4, 6, 39, and 18 SNPs for each exposure in the analysis. As shown in **Figure 2**, the IVW analysis results revealed that the presence of hypothyroidism was associated with a 75.8% higher risk of RA (OR=1.758, P=7.61E-05). The causal relationships between GD, other types of hyperthyroidism, TSH, FT4, and RA were insignificant (**Table 1**). The scatter plot for effect sizes of SNPs for each phenotype in the forward analysis is shown in **Figure 3**. Apart from GD (MR Egger intercept value is 0.1376, P=0.018), no horizontal pleiotropy was observed for other phenotypes. While TSH-related SNPs showed no heterogeneity after removing palindromic SNPs, substantial heterogeneity was observed in the final analysis of other phenotypes (**Table 2**). Therefore, we applied the IVW fixed-effects model for the final analysis of TSH and the random-effects model for the remaining exposures.

**Table 1:** MR results for the relationship between thyroid function and RA.

Exposure	Method	nSNP	F	OR (95% CI)	P
The relationship between hypothyroidism and RA (finn-b-E4_HYTHYNAS)					
finn-b-E4_HYTHYNAS	IVW	34	36861	1.758 (1.329,2.325)	7.61E-05
	MR Egger			2.569 (1.354,4.874)	0.007
	Weighted median			1.074 (0.976,1.181)	0.144
The relationship between GD and RA (finn-b-E4_THYTOXGOITDIF)					
finn-b-E4_THYTOXGOITDIF	IVW	4	709	0.928 (0.398,2.162)	0.863
	MR Egger			0.582 (0.069,4.941)	0.646
	Weighted median			0.899 (0.785,1.030)	0.126
The relationship between other types of hyperthyroidism and RA (finn-b-E4_THYTOXNAS)					
finn-b-E4_THYTOXNAS	IVW	6	22141	0.818 (0.618,1.083)	0.161
	MR Egger			0.502 (0.434,0.582)	0.012
	Weighted median			0.996 (0.906,1.095)	0.934
The relationship between TSH and RA					
TSH	IVW	39	3490	1.018 (0.935,1.109)	0.676
	MR Egger			1.142 (0.920,1.419)	0.237
	Weighted median			1.036 (0.929,1.155)	0.522
The relationship between FT4 and RA					
FT4	IVW	18	1012	1.076 (0.897,1.291)	0.432
	MR Egger			1.305 (0.786,2.164)	0.318
	Weighted median			1.218 (1.017,1.459)	0.032

All statistical tests were two-sided. P < 0.05 was considered significant.

nSNP, number of single nucleotide polymorphisms; OR, odds ratio; CI, confidence interval; IVW, inverse variance weighted; RA, rheumatoid arthritis; GD, Graves’ disease; FT4, free thyroxine; TSH, thyroid-stimulating hormone.

**Table 2:** The heterogeneity and sensitivity results of RA and thyroid function after removal of pleiotropic IVs.

Exposure-outcome	nSNP	MR Egger intercept		Cochran’s heterogeneity test			
		Intercept value	P	IVW-Q value	P (IVW)	Egger-Q value	P (Egger)
Hypothyroidism-RA	34	-0.0469	0.207	1058.7397	4.11E-201	1006.5788	7.05E-191
GD-RA	4	0.1376	0.018	53.7322	1.28E-11	1.0231	0.600
other types of hyperthyroidism-RA	6	0.1532	0.661	1057.9187	1.73E-226	1001.8007	1.45E-215
TSH-RA	39	-0.0080	0.267	50.5121	0.084	48.8353	0.092
FT4-RA	18	-0.0125	0.434	35.9153	0.005	34.5283	0.005
RA-Hypothyroidism	69	-0.0148	0.127	505.025	5.40E-68	469.202	1.09E-61
RA-GD	73	0.0276	0.067	365.270	8.23E-41	348.305	3.41E-38
RA-other types of hyperthyroidism	78	0.0234	0.081	303.373	1.27E-28	285.143	5.91E-26
RA-TSH	69	-0.0034	0.143	86.6897	0.063	83.9360	0.079
RA-FT4	70	0.0021	0.367	78.982	0.193	78.034	0.190

MR, Mendelian randomization; IVW, inverse variance-weighted; nSNP, number of single nucleotide polymorphisms; IVW, inverse variance weighted; RA, rheumatoid arthritis; GD, Graves’ disease; FT4, free thyroxine; TSH, thyroid-stimulating hormone.

### Influence of genetically predicted RA on thyroid function

**Figure 4:**
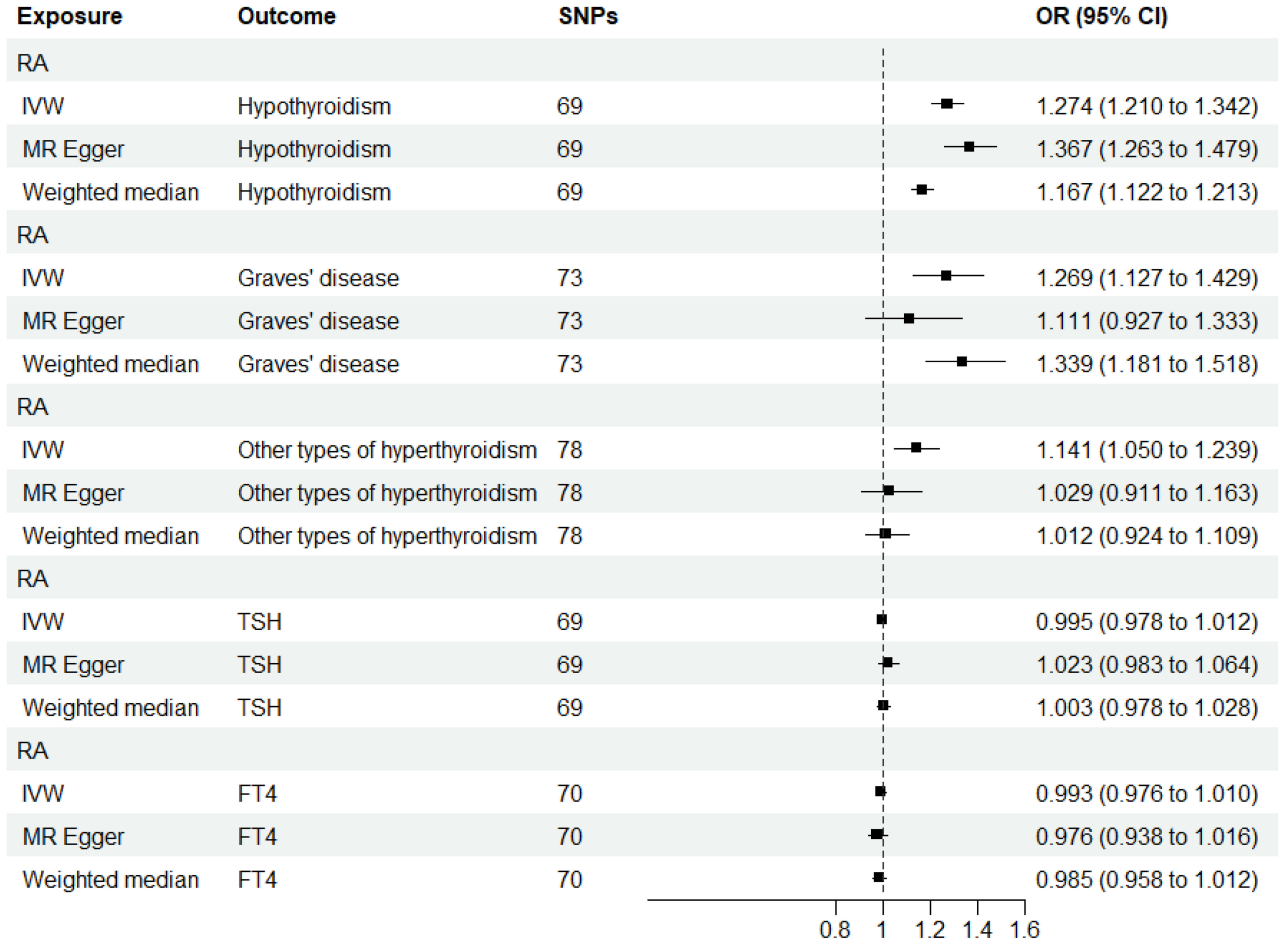
MR estimates for the causal effect of RA on thyroid function. IVW, inverse variance weighted; SNP, single-nucleotide polymorphism; OR, odds ratio; CI, confidence interval; RA, rheumatoid arthritis; TSH, thyroid-stimulating hormone; FT4, free thyroxine.

**Figure 5:**
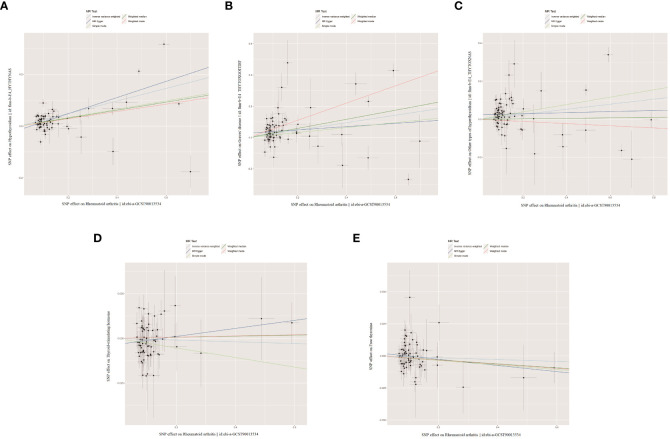
The scatter plots for the causal effect of reverse MR analysis. **(A)** Rheumatoid arthritis on hypothyroidism. **(B)** Rheumatoid arthritis on Graves' disease. **(C)** Rheumatoid arthritis on other types of hyperthyroidism. **(D)** Rheumatoid arthritis on thyroid-stimulating hormone. **(E)** Rheumatoid arthritis on free thyroxine.

When RA was considered as the exposure, after removing palindromic and heterozygous SNPs, we included 69, 73, 78, 69, and 70 SNPs for hypothyroidism, GD, other types of hyperthyroidism, TSH, and FT4, respectively, in the final analysis (excluded SNPs are listed in **Supplementary Table 8**). As shown in **Figure 4**, the IVW results indicated that the presence of RA was associated with a 27.4%, 26.9%, and 14.1% higher risk of hypothyroidism, GD, and other types of hyperthyroidism, respectively (OR=1.274, 1.269, 1.141, P=3.88x10^-20, 8.15x10^-5, 1.80x10^-3). The causal relationships between RA and TSH, RA and FT4, were insignificant using all three analysis methods (**Table 3**). The scatter plot for effect sizes of SNPs for each phenotype in the reverse analysis is shown in **Figure 5**. There was no evidence of horizontal pleiotropy based on the MR-Egger intercept tests. Significant heterogeneity was observed in the final analysis of RA and hypothyroidism, GD, and other types of hyperthyroidism (**Table 2**), and a random-effects model was employed. The IVW fixed-effects model was used to analyze RA and TSH, RA and FT4.

**Table 3:** MR results for the relationship between RA and thyroid function.

Exposure	Methods	nSNP	F	OR (95% CI)	P
The relationship between RA and hypothyroidism (finn-b-E4_HYTHYNAS)					
RA	IVW	69	48735	1.274 (1.210,1.342)	3.88E-20
	MR Egger			1.367 (1.263,1.479)	6.30E-11
	Weighted median			1.167 (1.122,1.213)	1.36E-14
The relationship between RA and GD (finn-b-E4_THYTOXGOITDIF)					
RA	IVW	73	49541	1.269 (1.127,1.429)	8.15E-05
	MR Egger			1.111 (0.927,1.333)	2.59E-01
	Weighted median			1.339 (1.181,1.518)	5.16E-06
The relationship between RA and other types of hyperthyroidism (finn-b-E4_THYTOXNAS)					
RA	IVW	78	77113	1.141 (1.050,1.239)	1.80E-03
	MR Egger			1.029 (0.911,1.163)	6.49E-01
	Weighted median			1.012 (0.924,1.109)	7.94E-01
The relationship between RA and TSH.					
RA	IVW	69	19336	0.995 (0.978,1.012)	0.577
	MR Egger			1.023 (0.983,1.064)	0.273
	Weighted median			1.003 (0.978,1.028)	0.815
The relationship between RA and FT4.					
RA	IVW	70	19756	0.993 (0.976,1.010)	0.409
	MR Egger			0.976 (0.938,1.016)	0.245
	Weighted median			0.985 (0.958,1.012)	0.271

All statistical tests were two-sided. P < 0.05 was considered significant.

nSNP, number of single nucleotide polymorphisms; OR, odds ratio; CI, confidence interval; IVW, inverse variance weighted; RA, rheumatoid arthritis; GD, Graves’ disease; TSH, thyroid-stimulating hormone; FT4, free thyroxine.

The leave-one-out analysis and visualization results demonstrate the robustness of our findings. The forest plots, leave-one-out sensitivity plots, and funnel plots of the present study are shown in **Supplementary Figures 1**–**6**.

## Discussion

This bidirectional two-sample MR study provides novel evidence of a bidirectional causal relationship between thyroid function and RA. The genetic susceptibility to hypothyroidism is associated with an increased risk of RA, while the genetic susceptibility to RA is associated with an increased risk of hypothyroidism, GD, and other types of hyperthyroidism. However, genetic susceptibility to GD and other types of hyperthyroidism is not associated to RA risk. Additionally, there is no evidence of a causal relationship, either forward or reverse, between FT4, TSH, and RA.

Our findings align with previous observational studies. The presence of thyroid dysfunction increases the risk of RA. A multicenter cross-sectional study involving 3,286 patients with autoimmune thyroid diseases revealed a significant elevation in the relative risk of other autoimmune diseases among individuals with GD or HT, with RA being the most prevalent (31). Similarly, a prospective cohort study in Italy involving 3,209 patients with GD found that 16.7% had another related autoimmune disease, with RA accounting for 1.9% (32). Conversely, the presence of RA increases the risk of thyroid dysfunction. A systematic review and meta-analysis conducted in 2022, which included 29 observational studies with 35,708 RA patients, demonstrated an increased risk of thyroid dysfunction among individuals with RA, particularly hypothyroidism (10).

Observational studies have been widely used to initially identify causal factors, but the unavoidable presence of reverse causation and potential confounding factors often limit the credibility (33). RCTs are considered the gold standard for causal inference in clinical research. However, they are often challenging to implement due to the time-consuming nature of large-scale follow-up and the high cost involved. MR methodology, utilizing genetic variants as IVs, offers a solution to the aforementioned challenges. Firstly, the random allocation of genetic variation at conception effectively avoids the influence of confounding factors (34). Secondly, the distribution of genotypes precedes exposure in time, ensuring that reverse causality does not affect the relationship between genotype and disease (35). Lastly, compared to the immediate outcomes obtained from RCTs, exposure factors obtained from a genetic standpoint often persist throughout a lifetime, mitigating the attenuation bias (regression dilution bias) (36).

A previous bidirectional MR study exploring the relationship between GD and RA in East Asian populations found that the presence of RA may increase the risk of GD by 39% (OR=1.39, 95% CI 1.10-1.75) (37). However, the relationship between genetic predisposition for GD and the risk of RA was not significant, consistent with our findings. Our study focuses on individuals of European ancestry and investigates the bidirectional causal relationships between other types of hyperthyroidism, FT4, TSH, and RA. It serves as a complementary and in-depth exploration of the MR findings by Wu et al. (37).

Genetic, immune, and environmental factors likely influence the correlation between RA and thyroid function, with genetic susceptibility playing a decisive role. Firstly, thyroid dysfunction commonly occurs in autoimmune thyroid diseases (AITD), particularly HT and GD. Autoimmune factors may be common features of thyroid autoimmunity and RA, with immune abnormalities, autoantibody production, immune cell activation, and release of inflammatory mediators potentially leading to the coexistence of these two diseases in certain patients (38–40). Secondly, environmental factors also play an essential role in the occurrence and development of hyperthyroidism, hypothyroidism, and RA. Specific environmental triggers (smoking, infections, medications, chronic stressors, Etc.) may induce or exacerbate the diseases by modulating the function of the immune system or by triggering disease occurrence through epigenetic mechanisms (41–44). Lastly, genetic factors majorly affect the interplay between hyperthyroidism, hypothyroidism, and RA. A positive family history is strong evidence for an increased risk of RA and AITD (45). Furthermore, multiple genes have been identified to be associated with susceptibility to RA and AITD, including the human leukocyte antigen DR B1 gene, the cluster of differentiation 226 gene, the protein tyrosine phosphatase non-receptor type 22, the Fc receptor-like 3 gene, the insulin-like growth factor 1 receptor gene, the cytotoxic T-lymphocyte-associated protein 4 gene, and the cluster of differentiation 40 gene, among others (37, 46). These genes have been proven to be involved in the disease processes of autoimmune thyroid diseases and RA through encoding relevant proteins, regulating cellular signaling, or participating in extensive pathways.

Our findings indicate that the association between hypothyroidism and RA is stronger compared to hyperthyroidism, consistent with most previous studies. In a cross-sectional study conducted by Mahagna et al., involving a cohort of 11,782 RA patients and 57,973 controls, it was found that RA posed a risk factor for both hyperthyroidism and hypothyroidism (OR=1.26, 1.42, P<0.001 for both). Additionally, the incidence of hypothyroidism among RA patients was higher than that of hyperthyroidism (16% vs. 2.3%) (9). ANOOP et al. reported that among their 100 RA patients, 22 had thyroid dysfunction, with hypothyroidism being the most common (15/22 cases) (47). Furthermore, a meta-analysis by Yinjin Liu et al., incorporating data from 29 studies involving a total of 35,708 RA patients, indicated that compared to hyperthyroidism (OR=1.64, 95% CI 1.64-2.19), RA risk was higher in hypothyroid patients (OR=2.25, 95% CI 1.78-2.84) (10). We speculate that this may be due to the lower immunogenicity associated with hyperthyroidism compared to the immunogenicity associated with hypothyroidism. Autoimmune-mediated thyroid dysfunction accounts for a higher proportion of hypothyroidism-related diseases than hyperthyroidism-related diseases (48). Thyroid dysfunction commonly occurs in AITD, particularly in HT and GD. The majority of existing research suggests that the mechanism behind thyroid dysfunction in RA patients is primarily attributed to the shared pathological pathways between the two conditions. Firstly, inflammatory mediators released in RA, such as interleukin-1, interleukin-6, and tumor necrosis factor-alpha, can act on thyroid tissues (49). Secondly, a disproportion in the ratio of T helper cells 17 to regulatory T cells in RA patients contributes to AITD (50). Additionally, as mentioned above, we speculate that genetics, epigenetics, and environmental factors may play crucial roles in the pathogenesis of the entire immune system. In addition, we selected the commonly used clinical markers, FT4 and TSH, for diagnosing thyroid dysfunction (51–53). The results demonstrate that neither hormone shows a causal relationship, either forward or reverse, with RA. We hypothesize that FT4 and TSH levels primarily reflect the secretory function of the thyroid gland and are not directly linked to the genetic, immune, and environmental mechanisms underlying the association between hyperthyroidism, hypothyroidism, and RA. This finding may indirectly suggest that the two hormones do not play a role in the genetic susceptibility mechanisms associated with RA, hyperthyroidism, and hypothyroidism. Alternatively, the limited sample size for the FT4 and TSH phenotypes could be a contributing factor.

This study has inherent limitations. Firstly, thyroid hormones, including multiple subtypes such as T3 and T4, were not fully explored in our bidirectional causal relationship with RA due to limitations in the available SNP data within the database. Consequently, we could not investigate the association between other thyroid hormone subtypes and RA. Secondly, there is a potential for horizontal pleiotropy in the analysis of GD and RA, which may impact our finding of no genetic susceptibility between GD and RA risk. Therefore, the result should be viewed cautiously. Thirdly, our study population was limited to individuals of European ancestry, and it remains to be seen whether our findings can be extrapolated to other populations.

## Conclusion

In conclusion, our study has revealed a bidirectional causal relationship between thyroid function and RA, providing new insights and evidence for understanding the etiology, screening, and management of comorbidity. Further research is needed to elucidate the underlying mechanisms linking thyroid function and RA and to validate this association through large-scale RCTs and scientific animal experiments.

## Data availability statement

The original contributions presented in the study are included in the article/**Supplementary Material**. Further inquiries can be directed to the corresponding author.

## Author contributions

PG and BP designed the study, wrote, reviewed, and edited the manuscript. DY and QX analyzed data. HL, TL, XZ, and CO reviewed and edited the manuscript. CO is the guarantor of this work. All authors contributed to the article and approved the submitted version.
