# Supplementary material for: Appraising the causal relationship between thyroid function and rheumatoid arthritis: a two-sample bidirectional Mendelian randomization study
Source: Front Immunol. 2023 Nov 28;14:1238757. doi: 10.3389/fimmu.2023.1238757 (PMC10713877; doi:10.3389/fimmu.2023.1238757)
Supplement: Supplementary file 1 [file DataSheet_1.docx]

***Supplementary Materials***

**SUPPLEMENTARY NOTES**

**Supplementary Figure 1 |** The forest plots for causal effect of thyroid function on RA.

**Supplementary Figure 2 |** The Leave-one-out sensitivity analysis for causal effect of thyroid function on RA.

**Supplementary Figure 3 |** The funnel plot of individual SNP effects of RA on thyroid function.

**Supplementary Figure 4 |** The forest plots for causal effect of RA on thyroid function.

**Supplementary Figure 5 |** The Leave-one-out sensitivity analysis for causal effect of RA on thyroid function.

**Supplementary Figure 6 |** The funnel plot of individual SNP effects of RA on thyroid function.

**Supplementary Figure 1 |** The forest plots for causal effect of thyroid function on RA.


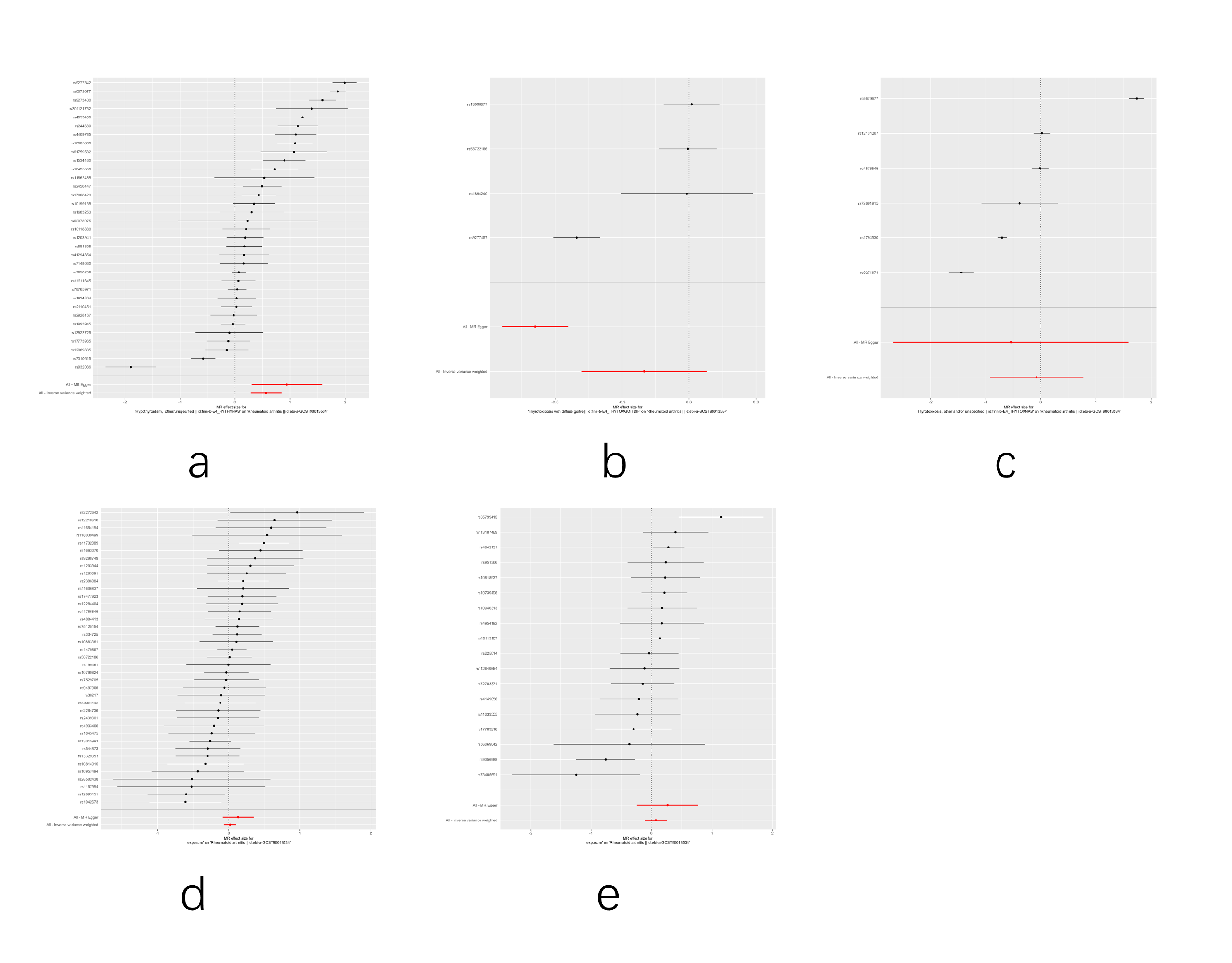


(a) hypothyroidism on RA, (b) hyperthyroidism with diffuse goitre on RA, (c) other unspecified hyperthyroidism on RA, (d) TSH on RA, (e) FT4 on RA.

**Supplementary Figure 2 |** The Leave-one-out sensitivity analysis for causal effect of thyroid function on RA.


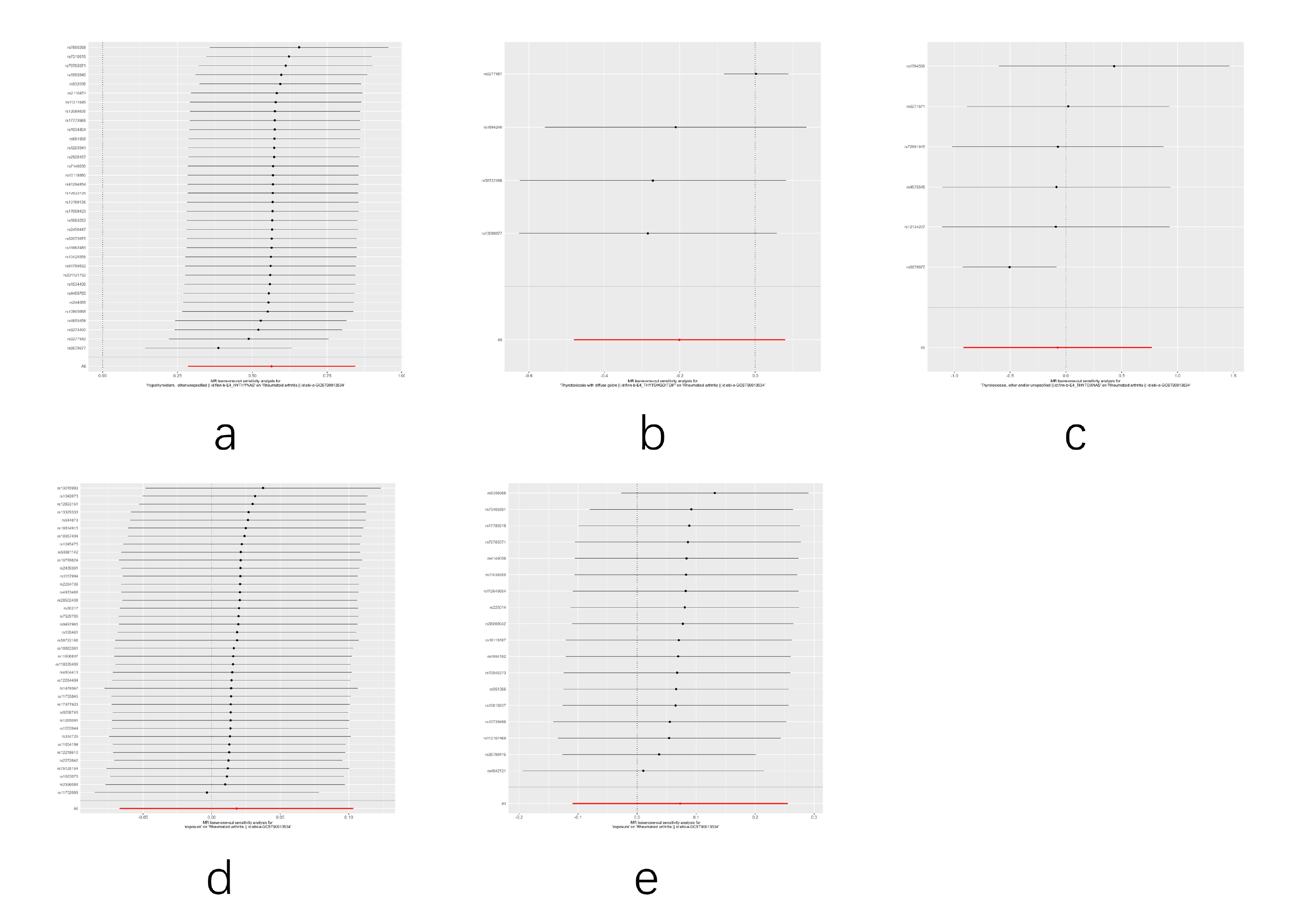


(a) hypothyroidism on RA, (b) hyperthyroidism with diffuse goitre on RA, (c) other unspecified hyperthyroidism on RA, (d) TSH on RA, (e) FT4 on RA.

**Supplementary Figure 3 |** The funnel plot of individual SNP effects of RA on thyroid function.


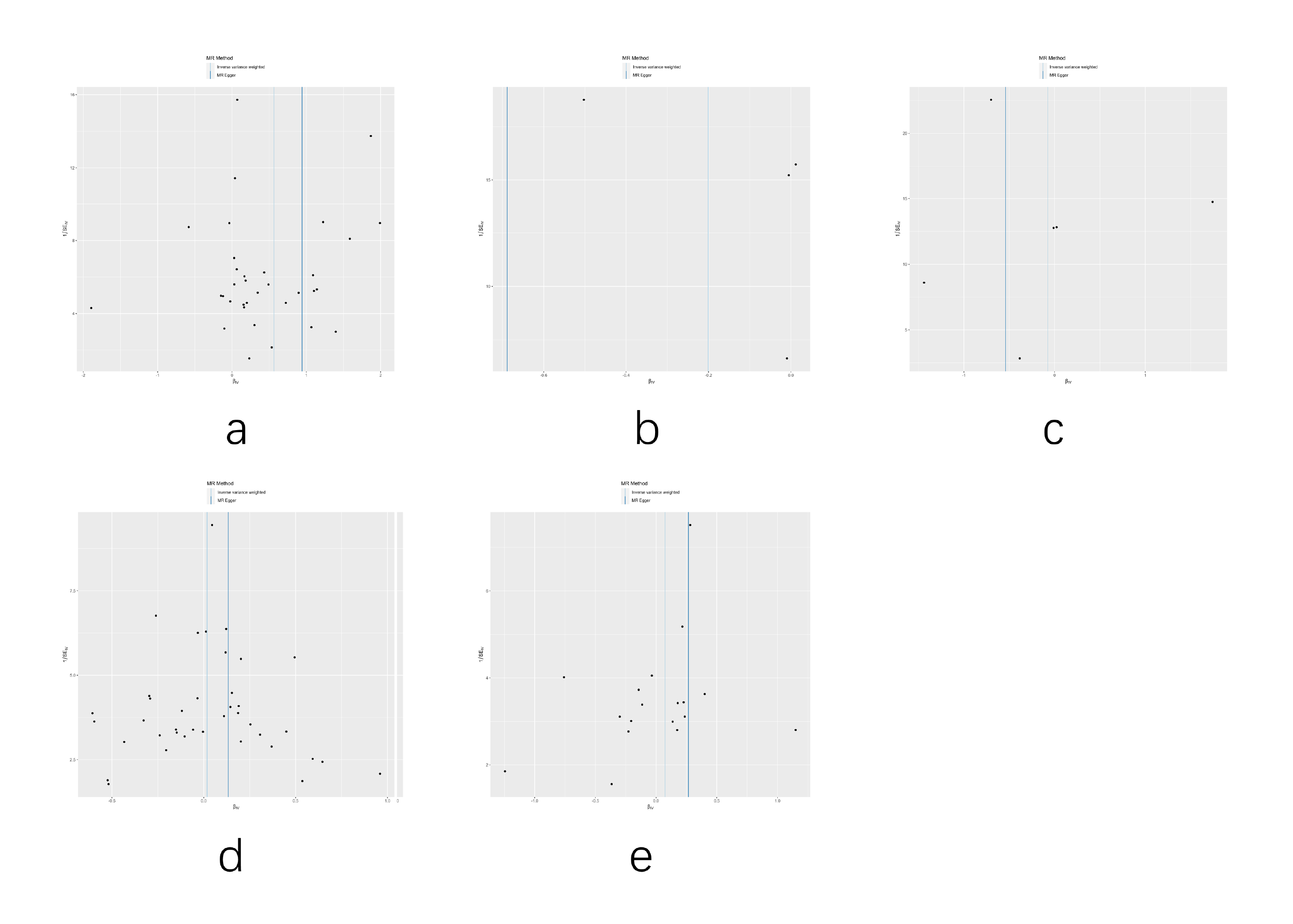


(a) hypothyroidism on RA, (b) hyperthyroidism with diffuse goitre on RA, (c) other unspecified hyperthyroidism on RA, (d) TSH on RA, (e) FT4 on RA.

**Supplementary Figure 4 |** The forest plots for causal effect of RA on thyroid function.


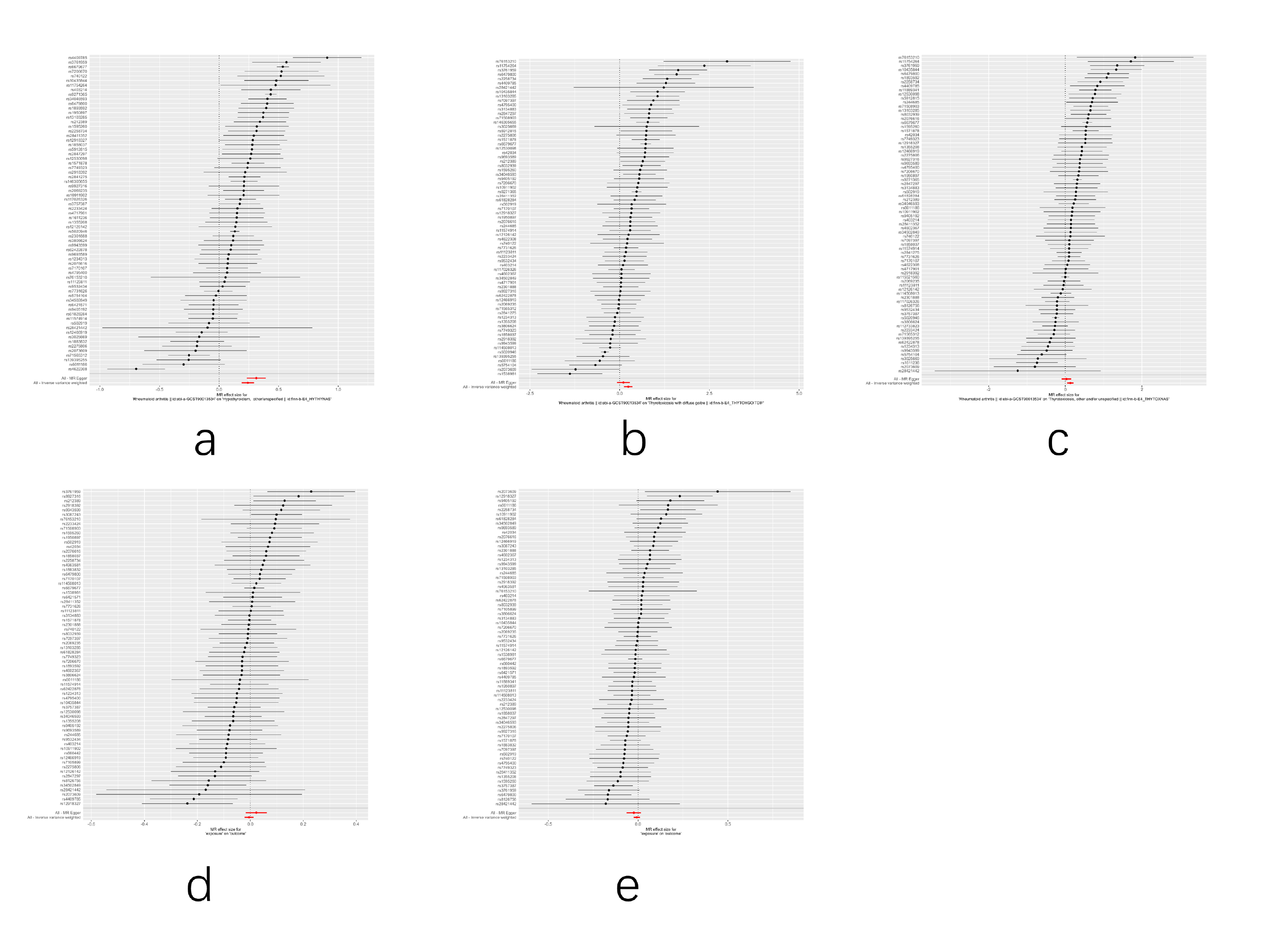


(a) RA on hypothyroidism, (b) RA on hyperthyroidism with diffuse goitre, (c) RA on other unspecified hyperthyroidism, (d) RA on TSH, (e) RA on FT4.

**Supplementary Figure 5 |** The Leave-one-out sensitivity analysis for causal effect of RA on thyroid function.


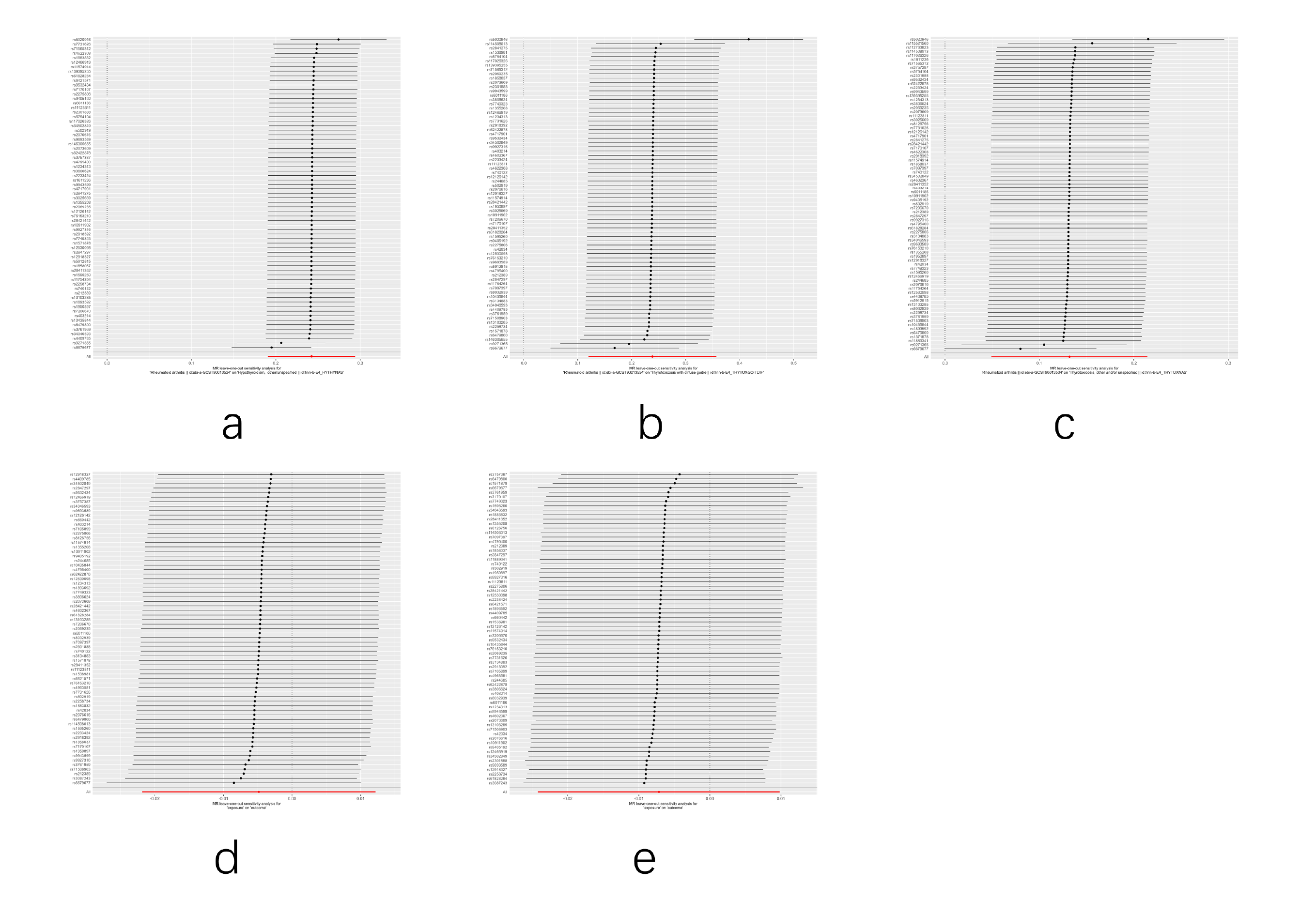


(a) RA on hypothyroidism, (b) RA on hyperthyroidism with diffuse goitre, (c) RA on other unspecified hyperthyroidism, (d) RA on TSH, (e) RA on FT4.

**Supplementary Figure 6 |** The funnel plot of individual SNP effects of RA on thyroid function.


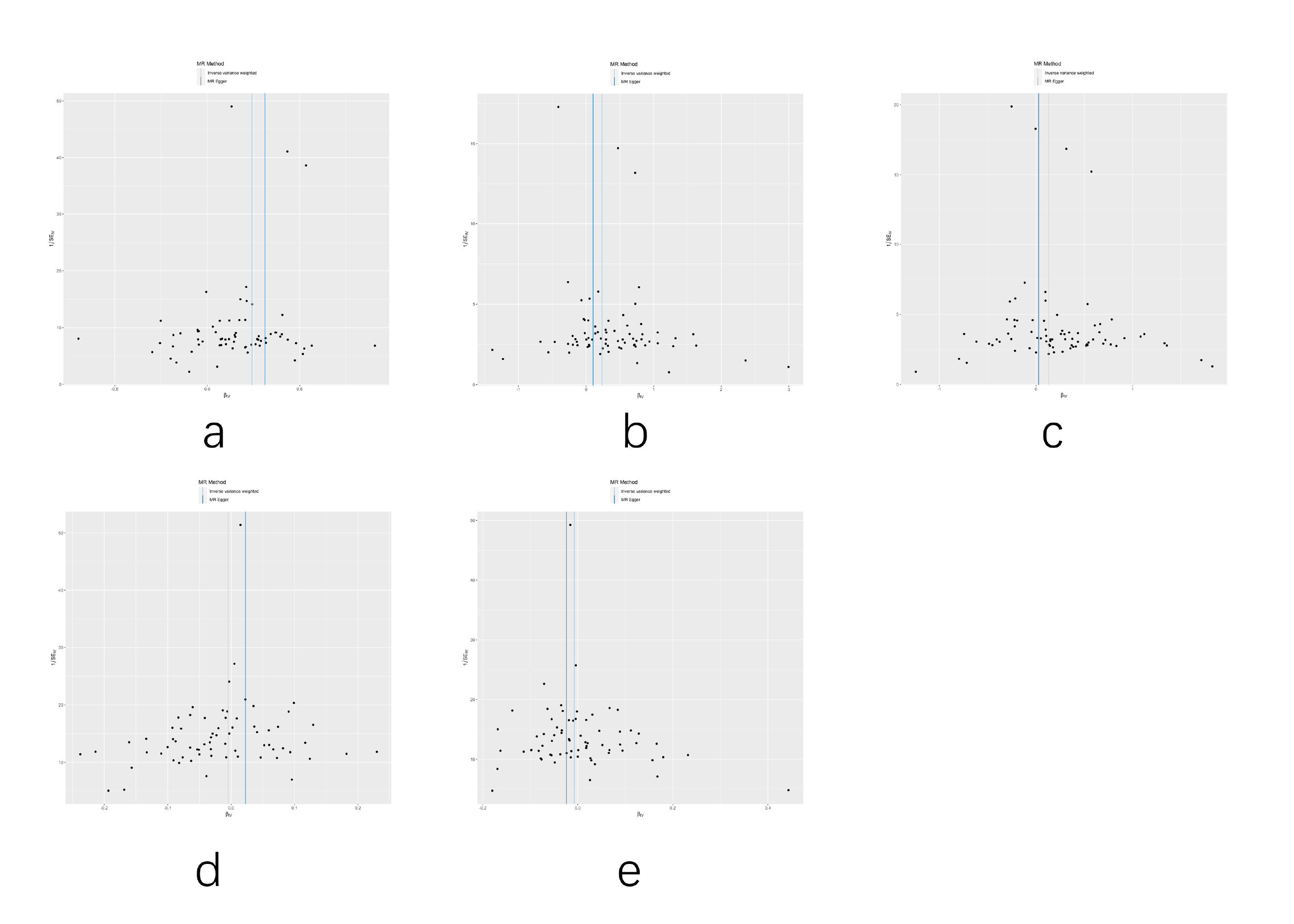


(a) RA on hypothyroidism, (b) RA on hyperthyroidism with diffuse goitre, (c) RA on other unspecified hyperthyroidism, (d) RA on TSH, (e) RA on FT4.
